# Improved Production of Cyclodextrins by Alkalophilic *Bacilli* Immobilized on Synthetic or Loofa Sponges

**DOI:** 10.3390/ijms131013294

**Published:** 2012-10-17

**Authors:** Tieles Carina de Oliveira Delani, Rúbia Pazzetto, Camila Sampaio Mangolim, Vanderson Carvalho Fenelon, Cristiane Moriwaki, Graciette Matioli

**Affiliations:** Department of Pharmacy, State University of Maringá (UEM), Maringá-PR 87020-900, Brazil; E-Mails: tielesfar@yahoo.com.br (T.C.O.D.); rubiapazzetto@yahoo.com.br (R.P.); camilamangolim@gmail.com (C.S.M.); vanderson2912@hotmail.com (V.C.F.); cmoriwaki@uem.br (C.M.)

**Keywords:** cyclodextrins, immobilization, loofa sponge, synthetic sponge, alkalophilic *Bacilli*

## Abstract

This study aimed to improve the production of β-cyclodextrin (β-CD) by microbial cells immobilized on synthetic or loofa sponges both with and without the use of alginate or chitosan. The most suitable matrix for the immobilization of *Bacillus firmus* strain 7B was synthetic sponge and for *Bacillus sphaericus* strain 41 was loofa sponge. After 330 days of storage, the β-CD production by *Bacillus firmus* and *Bacillus sphaericus* remained at around 41% and 49%, respectively, of initial levels. After 24 days of immobilization on loofa sponge, *Bacillus sphaericus* strain 41 achieved an improved operational stability, reaching 86.6 mM β-CD after 20 days of production, compared to only 32.8 mM of β-CD produced by free *Bacillus sphaericus* strain 41 cells. The expected increase in β-CD production by immobilized cells of *Bacillus firmus* strain 7B on synthetic sponge for 4 days was not statistically different to that for cells immobilized for 24 days. The application of this process on an industrial scale using loofa sponge, an inexpensive and renewable matrix, will allow the stable production of β-CD.

## 1. Introduction

Cyclodextrins (CDs) are cyclic oligosaccharides in which glucose units are linked by α,1–4 bonds. CDs possess the ability to encapsulate organic and inorganic molecules to form molecular inclusion complexes that modify the physicochemical properties of the guest molecule, increasing its stability and solubility [1,2]. A mixture composed mainly of α-CD, β-CD and a small amount of γ-CD is produced from a starch cyclization reaction catalyzed by CD glycosyltransferase (CGTase).

Alkalophilic *Bacilli* are more promising than other microorganisms for the production of CGTase because they show activity over wide pH and temperature ranges; they also produce β-CD in large quantities. β-CD is easily purified due to its solubility [3,4]. However, for extensive industrial use of cyclodextrins, their production cost has to be further reduced, which has prompted many researchers to seek new biotechnological processes for β-CD production. The immobilization of enzymes or CGTase-producing bacteria has been studied to achieve this goal [5–9].

The production of extracellular enzymes by immobilized cells has numerous advantages over production by free cells, such as the prolonged and repeated use of immobilized cells, easy separation of cells from the fermentation medium for continuous fermentation process in simple reactors and a reduced risk of contamination [9,10]. However, the method of immobilization should be chosen according to cell characteristics and mechanical properties of the matrix [11,12].

Two supports recently used in the immobilization of microbial cells are loofa sponge and synthetic sponge. The loofa sponge is a resistant matrix that displays high stability during use [13–17]. It has been reported as a support for the immobilization of cells using chitosan flocculants [18,19] and the entrapment of cells with calcium alginate gel [20]. Synthetic sponge has a polyurethane composition that is resistant and hydrophobic. When used as an immobilization support, it acts as an inert matrix and does not interfere with cell properties [21–24].

Although studies have reported the use of vegetable and synthetic sponges for cell immobilization, only one study has described using these two supports for the immobilization of alkalophilic *Bacilli* for CD biosynthesis [25]. An efficient application of this process on an industrial scale requires a technological system that is both stable and sustainable. The goal of this study is to achieve stability through repeated reuse of *Bacillus firmus* strain 7B and *Bacillus sphaericus* strain 41 cells immobilized on loofa or synthetic sponge.

## 2. Results and Discussion

### 2.1. β-Cyclodextrin Production by *Bacillus firmus* Strain 7B Cells Immobilized on Synthetic Sponge and *Bacillus sphaericus* Strain 41 Cells Immobilized on Loofa Sponge

**Figure 1:**
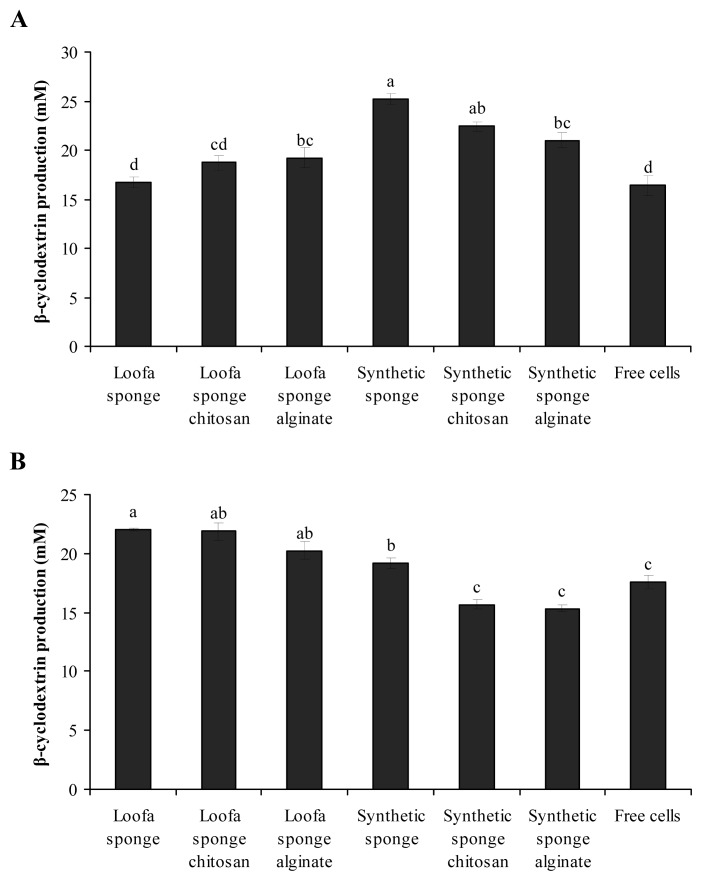
β-cyclodextrin (β-CD) production by (**A**) *B. firmus* strain 7B cells and (**B**) *B. sphaericus* strain 41 cells, free and immobilized on different matrices. Different letters in different columns represent statistically significant differences (*p* < 0.05). The same letters in different columns indicate no significant statistical difference.

Figure 1 shows β-CD production by cells of *B. firmus* strain 7B and *B. sphaericus* strain 41, both free and immobilized on loofa sponge, synthetic sponge, loofa sponge-chitosan and loofa sponge-alginate. β-CD production by *B. firmus* strain 7B cells (Figure 1A) was significantly higher when cells were immobilized in synthetic sponge (25.2 ± 0.5 mM) compared with immobilization in loofa sponge (16.7 ± 0.5 mM). β-CD production by cells immobilized on the loofa sponge-chitosan matrix (18.7 ± 0.8 mM) and loofa sponge-alginate matrix (19.2 ± 1.0 mM) was greater than for cells immobilized on loofa sponge without chitosan or alginate (16.7 ± 0.5 mM).

*B. firmus* strain 37 when immobilized on silica-titanium and silica-manganese showed that the initial β-CD production by immobilized bacteria on these matrices was lower [8]. The specific CGTase productivity showed a decrease when the initial biomass was raised for the immobilized *B. firmus* strain 37 cells in silica-titanium and silica-manganese, respectively, suggesting that biofilm layers are produced on the external surface of the matrix particles and that the activity is limited to only the biocatalyst located in the outer biofilm layer, which comes into contact with the substrate more easily [9]. This suggestion can be extrapolated to the matrices used in the current research.

In an effort to increase β-CD production, the synthetic sponge matrix was placed in the presence of chitosan, as *B. firmus* strain 7B cells showed a flocculation effectiveness of 94.3% for 10 mg chitosan/g cell; however, no increase in β-CD production was observed (22.4 ± 0.5 mM), demonstrating that the granules formed in the presence of chitosan may have limited the transfer of substrate through the matrix. This lack of transport may be a result of the small size of the pores of the synthetic sponge (Figure 1A). This is the first report of cells being immobilized on synthetic sponge in the presence of chitosan.

β-CD production by cells immobilized on a synthetic-alginate sponge matrix (21.0 ± 0.7 mM) was also lower than that of cells on synthetic sponge without alginate (25.2 ± 0.5 mM) (Figure 1A). The attempt to form an alginate gel around the synthetic sponge matrix to entrap a greater number of cells may have resulted in a decrease in the transfer of the enzyme CGTase to the fermentation medium and a subsequent decrease in β-CD production. Mazzer *et al*. [8] also found low β-CD production when *B. firmus* strain 37 was entrapped in beads of calcium alginate. Anisha and Prema [26], however, have shown good stability in the immobilization of *Streptomyces griseoloalbus* in alginate beads, along with increased α-galactosidase production. These studies show that the immobilization system may have very different behaviors depending on the microbial species being used and the enzyme being produced.

Synthetic sponge has been studied as a support for cell immobilization by other researchers [21,24], as it presents an inert structure that does not interfere in the fermentation processes in bioreactor, it is inexpensive and easily obtained. These properties make it a very attractive matrix for industries. Furthermore, Domíngues *et al*. [23] reported that synthetic sponge is suitable for immobilization because of its porosity, which improve the fixation of the cells and the transfer of oxygen and nutrients to the microorganism. Therefore, considering that in this study synthetic sponge matrix showed the best results for β-CD production in relation to different immobilization conditions, this matrix was chosen for further studies of the immobilization of *B. firmus* strain 7B.

β-CD production was measured for *B. sphaericus* strain 41 immobilized on the loofa and on synthetic sponge (Figure 1B). The flocculation effectiveness of this microorganism was 24.5%, which is inferior to that of *B. firmus* strain 7B. This result indicates that this alkalophilic bacillus has less chemical interaction among its cells in the presence of chitosan. No significant difference was observed between the β-CD production by *B. sphaericus* strain 41 cells immobilized on loofa sponge-chitosan matrix (21.9 ± 0.7 mM), loofa sponge-alginate (20.2 ± 0.7 mM) or loofa sponge matrix without chitosan or alginate (22.0 ± 0.2 mM) (Figure 1B).

For *B. sphaericus* strain 41, the use of the synthetic sponge matrix provided a β-CD concentration of 19.2 ± 0.4 mM, which is lower than that recorded for cells immobilized on loofa sponge matrix (22.0 ± 0.2 mM), on synthetic sponge-chitosan matrix (15.7 ± 0.4 mM) and on synthetic sponge-alginate matrix (15.3 ± 0.3 mM). This result demonstrated that neither chitosan nor alginate were able to improve the immobilization of *B. sphaericus* strain 41 on synthetic sponge (Figure 1B). Similar results have been previously reported for *B. firmus* strain 7B on synthetic sponge.

Considering the results obtained for β-CD production by *B. sphaericus* strain 41 cells in different matrices and by free cells, loofa sponge and loofa sponge-chitosan presented themselves as the best supports for immobilization, with no statistically significant difference between them. Thus, the loofa sponge matrix was chosen to continue the work, due to the low cost of reagents and the simplicity of the immobilization process.

### 2.2. Storage Stability

**Figure 2:**
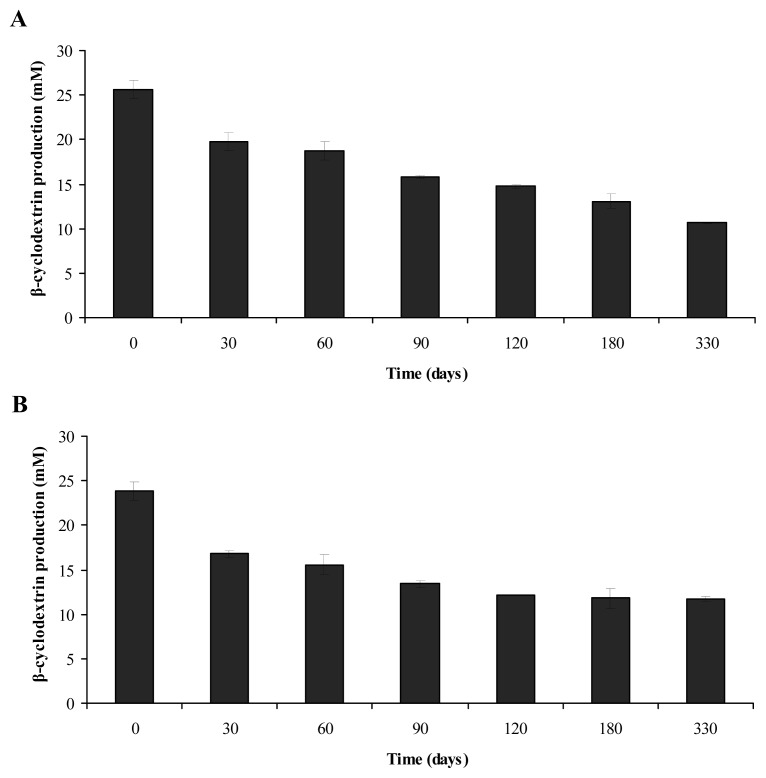
Storage stability in the β-CD production by (**A**) *B. firmus* strain 7B cells immobilized on a synthetic sponge matrix and (**B**) *B. sphaericus* strain 41 cells immobilized on a loofa sponge matrix.

One of the advantages of microbial cell immobilization is the ability to use them for a long period of time. The storage stability of immobilized cells was investigated under the same experimental conditions as above in the matrices on which cells produced highest levels of β-CD, *i.e.*, the synthetic sponge matrix for the immobilization of *B. firmus* strain 7B cells and loofa sponge matrix for the immobilization of *B. sphaericus* strain 41 cells. As expected, a gradual decrease in β-CD production was observed over 330 days (Figure 2). The cells immobilized on the synthetic sponge matrix were less stable than cells immobilized on the loofa sponge matrix, as the β-CD production by the microbial cells remained around 41% (Figure 2A) and 49% (Figure 2B), respectively, of initial values. Initially, the values obtained for production of β-CD were 25.6 ± 1.0 mM for cells immobilized on the synthetic sponge matrix (Figure 2A) and 23.8 ± 1.0 mM for cells immobilized on the loofa sponge matrix (Figure 2B). After 330 days, production decreased to 10.6 ± 0.0 mM and 12.7 ± 0.3 mM, respectively, showing that productivity was satisfactory during the period evaluated, compared with models of cell immobilization studied by other researchers.

Moriwaki *et al*. [9] found that storage stability for the *B. firmus* strain 37 in an inorganic matrix reached its maximum at 150 days, with approximately 4.4 mM β-CD; after 210 days, the amount of β-CD in the medium was less than 1.0 mM. While investigating the ethanol production in immobilized cell systems, Phisalaphong *et al*. [20] obtained a storage stability of 120 days.

### 2.3. Single Production Batch

**Figure 3:**
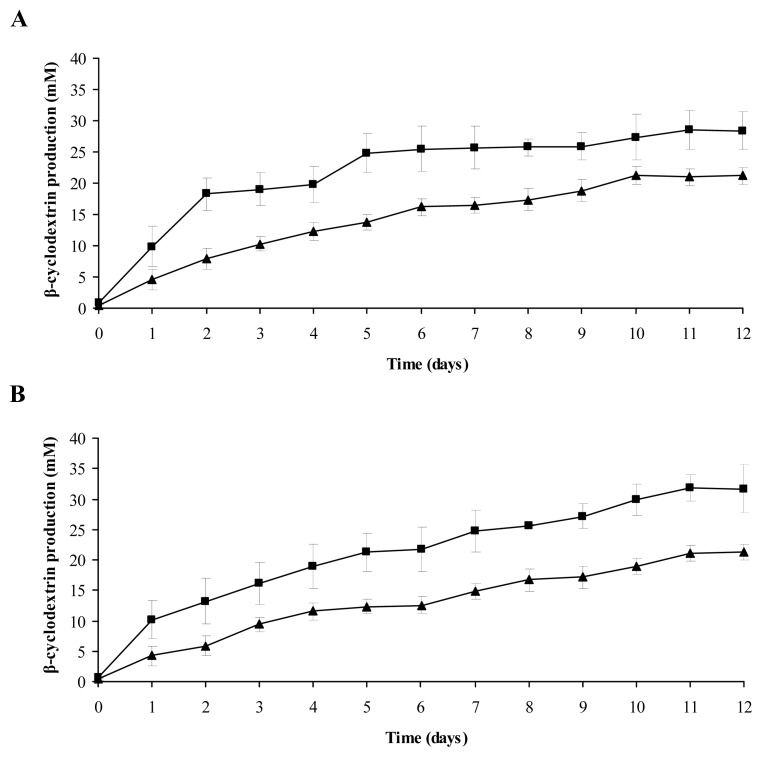
Single β-CD production batch over 12 consecutive days: (**A**) *B. firmus* strain 7B cells immobilized on a synthetic sponge matrix and (**B**) *B. sphaericus* strain 41 cells immobilized on a loofa sponge matrix; (■) immobilized cells and (▲) free cells.

β-CD production by free *B. firmus* strain 7B cells, *B. firmus* strain 7B cells immobilized on synthetic sponge matrix (Figure 3A), free *B. sphaericus* strain 41 cells and *B. sphaericus* strain 41 cells immobilized on loofa sponge matrix (Figure 3B) was evaluated in a single production batch, without changing the reaction medium for 12 consecutive days. A gradual increase in β-CD production up to 10 days was observed in both matrices and with free cells. β-CD production remained constant after this period. On the 12th day, cells immobilized on the synthetic sponge and loofa sponge matrices reached β-CD production levels of 28.4 ± 3.1 mM and 31.7 ± 3.9 mM, respectively. This profile of β-CD production was also found in a study on *B. firmus* strain 37 immobilized on silica-manganese and silica-titanium matrices and calcium alginate beads [8], as well as on a loofa sponge matrix [25]. Kunamneni *et al*. [6] observed a constant β-CD production by *Bacillus* sp. cells immobilized in alginate beads for 18 days, followed by decreased production after this time.

### 2.4. Operational Stability

**Figure 4:**
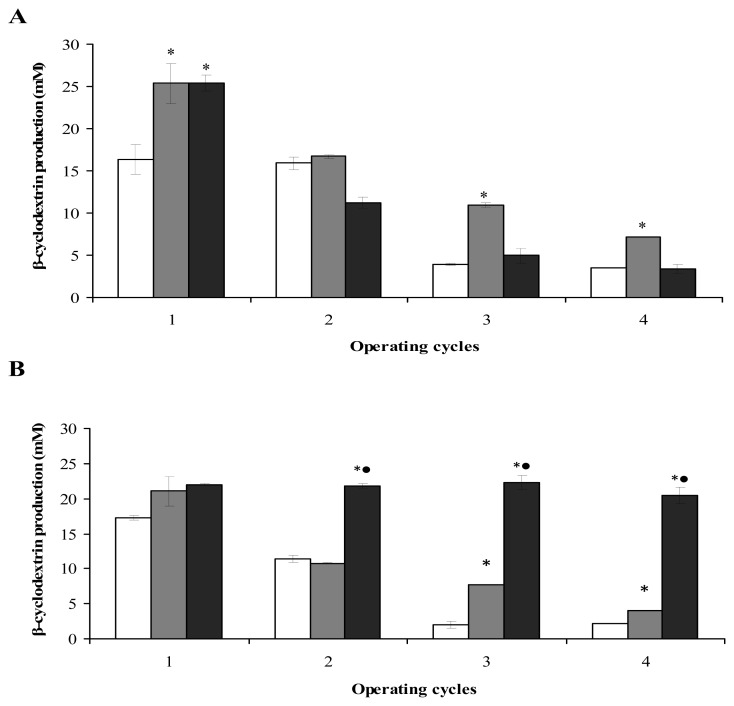
β-CD production in four consecutive cycles of 120 h each. (**A**) *B. firmus* strain 7B cells immobilized on a synthetic sponge matrix and (**B**) *B. sphaericus* strain 41 cells immobilized on a loofa sponge matrix; (□) free cells; (■) 4 days of immobilization; (■) 24 days of immobilization. * *P* < 0.05 when the β-CD production by the immobilized cells was compared with production by free cells. ^•^ *P* < 0.05 when β-CD production was compared between the immobilized cells for 4 and 24 days.

In this study, the reuse of *B. firmus* strain 7B cells immobilized on a synthetic sponge matrix (Figure 4A) and *B. sphaericus* strain 41 cells immobilized on a loofa sponge matrix (Figure 4B) were evaluated. In the first cycle, β-CD production by *B. firmus* strain 7B cells immobilized on synthetic sponge matrix was 25.4 ± 2.3 mM, 35.4% higher than for free cells (16.4 ± 1.7 mM). In the second, third and fourth cycles, β-CD production decreased to 66%, 43% and 28%, respectively, of levels in the first cycle. Production from free cells dropped dramatically to 3.9 ± 0.0 mM and 3.5 ± 0.0 mM β-CD in the third and fourth cycle, respectively.

The behavior of immobilized *B. firmus* strain 37 cells on loofa sponge was similar to that found by Pazzetto *et al*. [25]. Moldes *et al*. [24] immobilized *Phanerochaete chrysosporium* on nylon sponge and found a 35% decrease in the laccase production after the second operating cycle compared to the first cycle. Couto *et al*. [21] also observed a decrease in activity of the laccase produced by *Trametes hirsuta* immobilized on stainless steel sponge during the second cycle when compared to the first cycle.

The results obtained in this research showed a significant overall β-CD production at the end of four cycles for cells immobilized on the synthetic sponge matrix (60.2 mM) compared to production by free cells (39.7 mM), confirming the superiority of this immobilization system for *B. firmus* strain 37.

The best β-CD production by *B. sphaericus* strain 41 cells immobilized on loofa sponge matrix for 4 days was obtained in the first cycle (21.9 ± 2.1 mM); at this time, production was 21.5% higher than β-CD production by free cells (17.2 ± 0.2 mM). In the second, third and fourth cycles, β-CD production by immobilized cells remained higher than for free cells but gradually decreased. This decrease also was observed in other studies, such as with the clavulanic acid production by *Streptomyces clavuligens* immobilized on loofa sponge [27].

The total β-CD production by *B. sphaericus* strain 41 cells immobilized on loofa sponge matrix over four cycles was 44.5 mM compared with 32.8 mM for free cells. This result confirmed that immobilization was also a superior system for this microorganism.

Aiming to achieve greater operational stability and increased β-CD production, the *B. firmus* strain 7B cells and *B. sphaericus* strain 41 cells were immobilized for 24 days on synthetic or loofa sponge matrices, respectively. Production of β-CD was then evaluated for four consecutive cycles of 120 h each.

β-CD production by immobilized cells of *B. firmus* strain 7B for 4 days was not statistically different to that for cells immobilized for 24 days (Figure 4A); the expected increase in β-CD production was not observed. One hypothesis is that the synthetic sponge matrix did not provide a favorable environment for cellular renewal and the formation of additional colonies.

In addition, when the stability of *B. sphaericus* strain 41 cells immobilized on the loofa sponge matrix for 24 days was compared with the stability of cells immobilized for 4 days, there was a statistical difference in the second, third and fourth cycles. The 24-day immobilization procedure provided excellent operational stability, with β-CD production at around 22.0 ± 0.0 mM in the first, second and third cycles. In the fourth cycle, β-CD production remained at around 92% of the original level. At the end of the four cycles, total β-CD production was around 86.6 mM (Figure 4B).

Akhtar *et al*. [13] also obtained a high operational stability on removal and recovery of nickel from aqueous solution when *Chlorella sorokiniana* cells were immobilized on loofa sponge over a period of 24 days, allowing their repeated use for seven consecutive cycles without loss of cellular activity. Although other studies have also reported good operational stability of CD production by immobilized cells, the raw materials required are expensive and difficult to manufacture, resulting in a higher final price [6,28,29]. Additionally, few renewable resources were investigated in these prior studies. Using non-renewable resources could result in losses to the environment. The loofa sponge, on the order hand, is both biodegradable and environmentally friendly. The simple immobilization method detailed here could contribute to low operating costs for β-CD production on an industrial scale. Therefore, the results of this research demonstrated success in improving the immobilization of bacterial cells for CD biosynthesis.

Operational stability could be further studied by varying the time of cell immobilization within the range of 4 to 24 days and continuing operational cycles until the production of β-CD ceases. It would also be interesting to investigate the behavior of *B. firmus* strain 7B cells immobilized on loofa sponge for up to 4 days.

### 2.5. Scanning Electron Microscopy (SEM)

**Figure 5:**
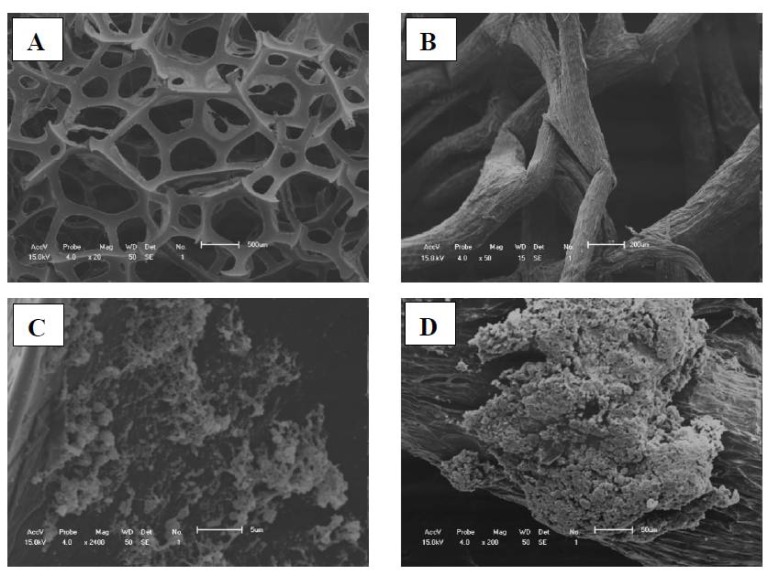
Scanning electron microscopy of synthetic sponge (**A**); loofa sponge (**B**); *B. firmus* strain 7B cells immobilized on synthetic sponge (**C**) and *B. sphaericus* strain 41 cells immobilized on loofa sponge (**D**).

The SEM images of synthetic and loofa sponge are shown in the Figure 5 A,B, respectively. The *B. firmus* strain 7B cells immobilized on the synthetic sponge matrix after 24 days are shown in Figure 5C, and *B. sphaericus* strain 41 cells immobilized on loofa sponge are shown in Figure 5D. In both matrices, immobilized cells in the form of granules were detected throughout the sponges, confirming the effectiveness of immobilization.

## 3. Experimental Section

### 3.1. Microorganisms and Culture Conditions

Immobilization of *B. firmus* strain 37 in different matrices has been previously reported [8,9,25]. The current work focuses on the effect of immobilization on alkalophilic microorganisms. *B. firmus* strain 7B and *B. sphaericus* strain 41 were isolated from the soil of planting oats [30] and soybean plants [31], respectively. These two microorganisms were grown on plates containing solid medium composed of (%, *w*/*v*) 1.0 soluble starch, 0.5 polypeptone, 0.5 yeast extract, 0.1 K_2_HPO_4_, 0.02 MgSO_4_·7H_2_O, 0.01 Congo red dye, 1.0 Na_2_CO_3_ and 1.5 agar. The plates were incubated at 37 °C for 48 h, and the colonies that formed were harvested from the middle and suspended in small amount of sterile saline (0.9% NaCl, *w*/*v*). Subsequently, 1 mL aliquots of the saline suspension were transferred to small glass vials, frozen and lyophilized (lyophilizer CHRIST—BETA 1–16). The lyophilized cells were stored in a −18 °C freezer before use in the immobilization procedures and experiments with free cells.

### 3.2. Microorganism Reactivation

To a 250 mL Erlenmeyer flask containing 50 mL of liquid medium was added 70 mg of *B. firmus* strain 7B or *B. sphaericus* strain 41 lyophilized cells. The liquid medium contained the same components as the solid medium, except the agar and dye were omitted. The flasks were incubated at 37 °C in an incubator with orbital shaking (120 rpm) for 24 h.

### 3.3. Cell Flocculation

Chitosan was used to induce the flocculation of cells according to the method of Ogbonna *et al*. [18] with some modifications. A cell suspension of optical density 600 nm (*A*) was added to a solution containing 1% chitosan (*w*/*v*) and 2% acetic acid (*v*/*v*) cells so that the final concentration of chitosan was 10 mg/g cells. After shaking, the mixture was stored for 1 h, and the optical density of the supernatant was measured (*B*). The effectiveness of flocculation was expressed as the percent reduction in the optical density according to the formula (*A* − *B*/*A*) × 100.

### 3.4. Cell Immobilization Procedure

The loofa sponge (*Luffa operculata*) and a synthetic sponge without antimicrobial chemicals (polyurethane, Scotch Brite™, 3M Company, Brazil) were used for immobilization of cells by adsorption. For the flocculation method, the loofa and synthetic sponge were positioned in the presence of chitosan. For the trapping method, they were positioned in the presence of calcium alginate. This method of immobilization by adsorption was developed by Meleigy and Khalaf [15] and modified by Pazzetto *et al*. [25].

In another experiment, cells were immobilized by adsorption over 24 days. During this time, the culture medium was changed five times according to the method developed by Akhtar *et al*. [13], with some modifications. *B. firmus* strain 7B and *B. sphaericus* strain 41 lyophilized cells (70 mg each) were weighed into liquid medium to reactivate for 24 h under the operating conditions described above. Following this, three discs of loofa or synthetic sponge were added to each flask. These remained in the middle for 4 days. The discs were then removed from the medium and placed in 100 mL of fresh medium, where they remained for over 5 days. This process was performed multiple times to produce a final immobilization time of 24 days.

Cells were also immobilized on loofa sponge chitosan or synthetic sponge chitosan by flocculation. The procedure used was the same as for the immobilization by adsorption, differing only in the addition of chitosan to the culture medium on the 3rd day. This procedure followed the methodology of Vignoli *et al*. [32] and Ogbonna *et al*. [18], with some modifications.

The immobilization of cells on loofa sponge-alginate and synthetic sponge-alginate matrices was done according to Phisalaphong *et al*. [20], with some modifications. A 3% alginate solution (*w*/*v*) was prepared by dissolving sodium alginate in 0.9% NaCl (*w*/*v*). After 4 days of culture in liquid medium, the cells were removed by centrifugation, resuspended in 5 mL of sterile 0.9% NaCl (*w*/*v*) and added to 50 mL of 3% alginate (*w*/*v*). To prepare the loofa sponge-alginate matrix and the synthetic sponge-alginate matrix, 3 discs of sterile sponge were immersed in the alginate-cell mixture. Sponges with gel were transferred to a solution of 1.47% CaCl_2_ (*w*/*v*) and gently agitated for 15 min. The matrices were then washed in a 0.9% NaCl (*w*/*v*) solution. The control for the cell immobilization assay consisted of culture medium and production medium containing only the free cells.

### 3.5. Cyclodextrin Production from Immobilized Microorganisms

After the immobilization procedures described above, the matrices were washed with 0.9% NaCl (*w*/*v*) and transferred to 250 mL Erlenmeyer flasks containing 50 mL of medium for CD production. This medium was composed of 10% maltodextrin (*w*/*v*) in 50 mM Tris-HCl buffer and 5 mM CaCl_2_, pH 8.0. β-CD production was conducted for 120 h at 60 °C in an incubator with shaking at 100 rpm. Aliquots of 1 mL were collected daily, diluted in 1 mL of distilled water and boiled for subsequent quantification of the β-CD produced.

Following the above methodology, the β-CD production was evaluated in a single production batch without changing the reaction medium for 12 consecutive days by free *B. firmus* strain 7B and *B. sphaericus* strain 41 cells, *B. firmus* strain 7B cells immobilized on synthetic sponge matrix and *B. sphaericus* strain 41 on loofa sponge matrix.

### 3.6. Operational Stability

β-CD was produced in cycles by *B. firmus* strain 7B cells immobilized on synthetic sponge and by *B. sphaericus* strain 41 cells immobilized on loofa sponge. Four cycles of 120 h were conducted. At the end of each cycle, sponges containing immobilized cells were removed from the medium and placed in fresh production medium. The assay was conducted according to the Section 3.5.

### 3.7. Storage Stability

To evaluate storage stability, cells of *B. firmus* strain 7B immobilized on synthetic sponge and *B. sphaericus* strain 41 immobilized on loofa sponge were prepared according to the immobilization procedures described above and stored at 4 °C for up to 330 days. At time points of 0, 30, 60, 90, 120, 180 and 330 days, the cells immobilized in loofa and synthetic sponge were transferred to medium, and CD production was measured as above.

### 3.8. Scanning Electron Microscopy (SEM)

SEM was performed according to the procedure described by Moriwaki *et al*. [9]. Cells immobilized on the synthetic and loofa sponges were placed in 50 mM Tris-HCl buffer and 5 mM CaCl_2_, pH 8.0, containing 2.5% glutaraldehyde for 24 h. Following this incubation, the supports containing the immobilized cells were washed with solutions of 30%, 50%, 70%, 90% and 100% ethanol. The material remained in absolute ethanol for further dehydration in an extraction system using supercritical CO_2_ under high pressure. To obtain the micrographs, a scanning electron microscope (Shimadzu model—SS 550) was used with an acceleration voltage of 10 kV.

### 3.9. Determination of β-Cyclodextrin

β-CD concentration was determined with a colorimetric assay in which the absorption of phenolphthalein at 550 nm changes after complexation with β-CD [8,25,31]. The assay was carried out by mixing 0.5 mL of the sample containing β-CD with 2.5 mL of 0.06 mM phenolphthalein working solution and 0.12 M bicarbonate-carbonate buffer, pH 10.5. For a blank, the sample was replaced with distilled water. The phenolphthalein working solution was prepared at the time of dosing from a 3 mM phenolphthalein stock solution in 95% ethanol.

### 3.10. Statistical Analysis

The immobilization assay results for different matrices and free cells were submitted to analysis of variance (ANOVA) and the Tukey test, with significance set at 5%. For the operational stability results, Student’s *t*-test was used with a 5% significance level. Both tests were carried out using Statistica 8.0/2008 (Stat Soft, Inc. Tulsa, OK, USA).

## 4. Conclusions

The best matrices for the immobilization of *B. firmus* strain 7B and *B. sphaericus* strain 41 cells were synthetic and loofa sponges, respectively, without alginate or chitosan. Using these matrices for immobilization, these cells showed acceptable storage stability and continuous production of β-CD. *B. sphaericus* strain 41 cells immobilized on loofa sponge achieved an improved operational stability after 20 days of production, compared to β-CD produced by free cells.

Due to the low cost and renewable character of loofa sponge, this research conducted on an industrial scale, will enable an appropriate β-CD production.
